# Editorial: Community series in the immunological role of the maternal microbiome in pregnancy, Volume II

**DOI:** 10.3389/fimmu.2026.1872195

**Published:** 2026-05-18

**Authors:** Nicoletta Di Simone, Eytan R. Barnea, Martin Mueller

**Affiliations:** 1 Department of Biomedical Sciences, Humanitas University, Milan, Italy; 2 Humanitas San Pio X, Milan, Italy; 3 Society for the Investigation of Early Pregnancy (SIEP), New York, NY, United States; 4 Department of Obstetrics, Gynecology & Reproductive Sciences, University of Miami Miller School of Medicine, Miami, FL, United States; 5 Department of Obstetrics, Lindenhofgruppe AG, Bern, Switzerland; 6 Institute of Biochemistry and Molecular Medicine, University of Bern, Bern, Switzerland

**Keywords:** endometrium, inflammation, microbiome, microbiota, pregnancy

The notion of a sterile uterine environment has been progressively overturned. Evidence now indicates that the endometrium hosts biologically relevant microbiota, whose role—although still debated—appears increasingly central to reproductive success. At the center of this emerging paradigm lies the intricate crosstalk between the endometrial microbiota and the maternal immune system. It is not simply the presence of microbes, but their functional integration within the immunological and endocrine landscape that influences implantation and early pregnancy.

Alterations in endometrial microbial composition have been associated with recurrent implantation failure (RIF) following embryo transfer and with spontaneous miscarriage. A recurrent observation is the depletion of *Lactobacillus* species accompanied by an increased abundance of facultative and obligate anaerobes. These microbial shifts are thought to disrupt embryo–endometrium signaling and promote a pro-inflammatory or immunologically dysregulated state, ultimately compromising implantation and early placental development (Gao et al.).

Maternal factors, including obesity, further shape this microbial landscape. In assisted reproduction, women with higher body mass index exhibit distinct uterine microbiota profiles—characterized by reduced *Lactobacillus* and increased pathogenic taxa—paralleling lower implantation and pregnancy rates. These findings reinforce the view that metabolic and microbial pathways are closely intertwined (Yan et al.).

This perspective extends across pregnancy. A stable, *Lactobacillus*-dominated vaginal microbiota is consistently associated with term delivery, whereas increased diversity and depletion of protective species are linked to preterm birth. Recent studies suggest that this risk is not only microbial but also metabolic: specific cervicovaginal metabolite profiles, alongside microbial shifts, precede preterm delivery, pointing toward a functional, rather than purely compositional, disruption (Wang et al.).

What emerges from this body of work is a shift from viewing the microbiota as a passive marker to considering it an active participant in reproductive biology. This shift carries clinical implications, although translation remains at an early stage. Microbiota profiling and integrated metabolomic approaches hold promise for identifying women at risk of implantation failure, miscarriage, preterm birth, and pregnancy complications. At the same time, interventions aimed at modulating the microbiota—through diet, probiotics, or more targeted strategies—are being explored, albeit without definitive evidence for routine use.

In this evolving landscape, the critical question is how the microbiota’s complexity can be harnessed to inform next-generation diagnostics and therapeutics. Translation into clinical practice demands caution. The field remains constrained by technical variability, low microbial biomass, susceptibility to contamination, and the enduring challenge of distinguishing causation from correlation. Rigorous standardization, longitudinal designs, and mechanistic studies will be essential to bridge this gap.

The question is no longer whether the maternal microbiota matters, but how its complexity can be translated into clinically meaningful advances.

